# CRISPaint allows modular base-specific gene tagging using a ligase-4-dependent mechanism

**DOI:** 10.1038/ncomms12338

**Published:** 2016-07-28

**Authors:** Jonathan L. Schmid-Burgk, Klara Höning, Thomas S. Ebert, Veit Hornung

**Affiliations:** 1 Institute of Molecular Medicine, University Hospital, University of Bonn, Sigmund-Freud-Strasse 25, 53127 Bonn, Germany; 2 Gene Center and Department of Biochemistry, Ludwig-Maximilians-Universität München, Feodor-Lynen-Strasse 25, 81377 Munich, Germany; 3 Center for Integrated Protein Sciences, Munich, Germany

## Abstract

The site-specific insertion of heterologous genetic material into genomes provides a powerful means to study gene function. Here we describe a modular system entitled CRISPaint (CRISPR-assisted insertion tagging) that allows precise and efficient integration of large heterologous DNA cassettes into eukaryotic genomes. CRISPaint makes use of the CRISPR-Cas9 system to introduce a double-strand break (DSB) at a user-defined genomic location. A universal donor DNA, optionally provided as minicircle DNA, is cleaved simultaneously to be integrated at the genomic DSB, while processing the donor plasmid at three possible positions allows flexible reading-frame selection. Applying this system allows to create C-terminal tag fusions of endogenously encoded proteins in human cells with high efficiencies. Knocking out known DSB repair components reveals that site-specific insertion is completely dependent on canonical NHEJ (DNA-PKcs, XLF and ligase-4). A large repertoire of modular donor vectors renders CRISPaint compatible with a wide array of applications.

## Abstract (web summary)

The use of site-specific gene insertion is a powerful method for investigating gene function. Here the authors describe CRISPaint, a universal tagging system using CRISPR-Cas9 to insert genes in an NHEJ dependent manner.

## Graphical abstract

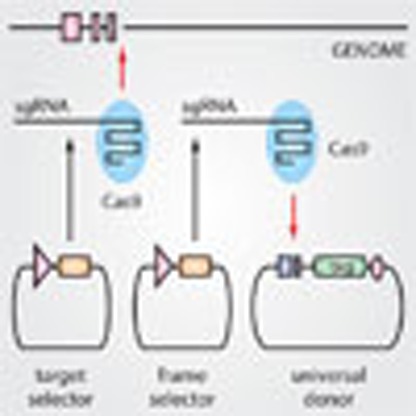

The development of custom-designed Zinc Finger^1^, TALE^2^ and RNA-guided^3^,^4^,^5^ nucleases has opened the possibility of cutting eukaryotic genomes at user-defined positions with high specificity. In this respect, RNA-guided nucleases of the CRISPR-Cas9 family currently provide the most convenient genome engineering toolbox, since they are simple to use, with only a single variable 17-20-mer RNA sequence dictating their target specificity^3^,^6^,^7^,^8^,^9^,^10^. When expressed in cells, designer nucleases introduce double-strand breaks (DSB) in genomic DNA, which cells strive to repair to retain their ability to replicate. To this effect two major repair pathways have been described that are able to repair DSBs: non-homologous end joining (NHEJ) and homology directed repair (HDR)^11^,^12^,^13^. While HDR requires a sister chromatid or a homologous chromosome as a blueprint for scarless repair, NHEJ does not rely on a homologous template, yet comprises the re-ligation of the ends of the DSB. While in principle faithful, NHEJ pathways can result in random mutagenesis at the site of the DSB, especially in the presence of incompatible overhangs or blocked ends^11^,^12^. NHEJ can proceed into two distinct pathways: while the canonical NHEJ (cNHEJ) machinery re-ligates DSBs employing ligase-4 without or with only minimal modification of the respective dsDNA ends, alternative NHEJ (aNHEJ) directs the re-ligation of resected ends of DSBs at positions of microhomologies, that is, homologous sequence stretches that are only a few bases long^11^. Due to this preceding end-resection, aNHEJ inherently leads to a loss of genetic information.

Cellular repair pathways can be exploited to manipulate the genomic architecture. For the generation of mammalian knockout alleles, it usually suffices to disrupt the reading frame of a gene by introducing a DSB into its coding region, which results in indel mutagenesis largely mediated by cNHEJ (ref. 14), but also associated with aNHEJ (ref. 15) repair pathways.

On the other hand, introduction of heterologous genetic material at a specific site is more challenging: classically, HDR is used to integrate a DNA fragment of choice with homology arms into the genome. Since DSB induction dramatically induces HDR (ref. 16), short homology arms^17^ and even single-stranded DNA oligonucleotides can be used for this purpose^18^. In this regard, it has also been reported that during HDR-mediated DNA insertion, a fraction of cells contains integrants resulting from homology-dependent recombination at only one homology arm, whereas the other end of the donor DNA integrates in a non-sequence specific manner^19^,^20^,^21^. At the same time, genetic material can also be introduced at the site of a DSB independently of HDR, just by providing short microhomologies between the target and the donor DNA^22^, or by utilizing cNHEJ mechanisms, either using synthetic DNA with pre-formed overhangs complementary to the DSB (ref. 17) or using plasmid DNA that is cleaved within the recipient cells by a designer nuclease^23^,^24^,^25^,^26^,^28^. Along these lines, it has also been demonstrated that blunt-end double-stranded DNA oligonucleotides with phosphothioate modifications are precisely inserted into genomic lesions via mechanisms suggestive of cNHEJ (ref. 29). HDR-independent integration methods hold the promise of providing a simple and modular approach to targeted genome engineering. However, their sequence-dependence, targeting range, efficiency and fidelity in mammalian cells still remain to be explored before a general concept for their applicability can be provided.

Based on these considerations, we strived to develop a simple and modular methodology that would allow the precise integration of heterologous genetic material at user-defined genomic locations. This technique should be efficient so that no selection cassette would be required, and at the same time it should not require any time-consuming preparatory steps, for example, the cloning of donor templates. Moreover, only minor targeting constraints should limit its applicability for the insertion of heterologous genetic material at user-defined locations. Amongst many possible applications, such a system would be of great use for the tagging of endogenous genes so that C-terminal fusions of respective proteins would be expressed, allowing their quantification and visualization at the endogenous level in living cells.

The method developed here provides an efficient and precise route to tag gene insertion into endogenous protein coding genes in mammalian cells. Through relying on the cellular NHEJ-machinery, the method does not require the preparation of target-specific homologous DNA constructs.

## Results

### First-generation modular gene tagging

**Figure 1:**
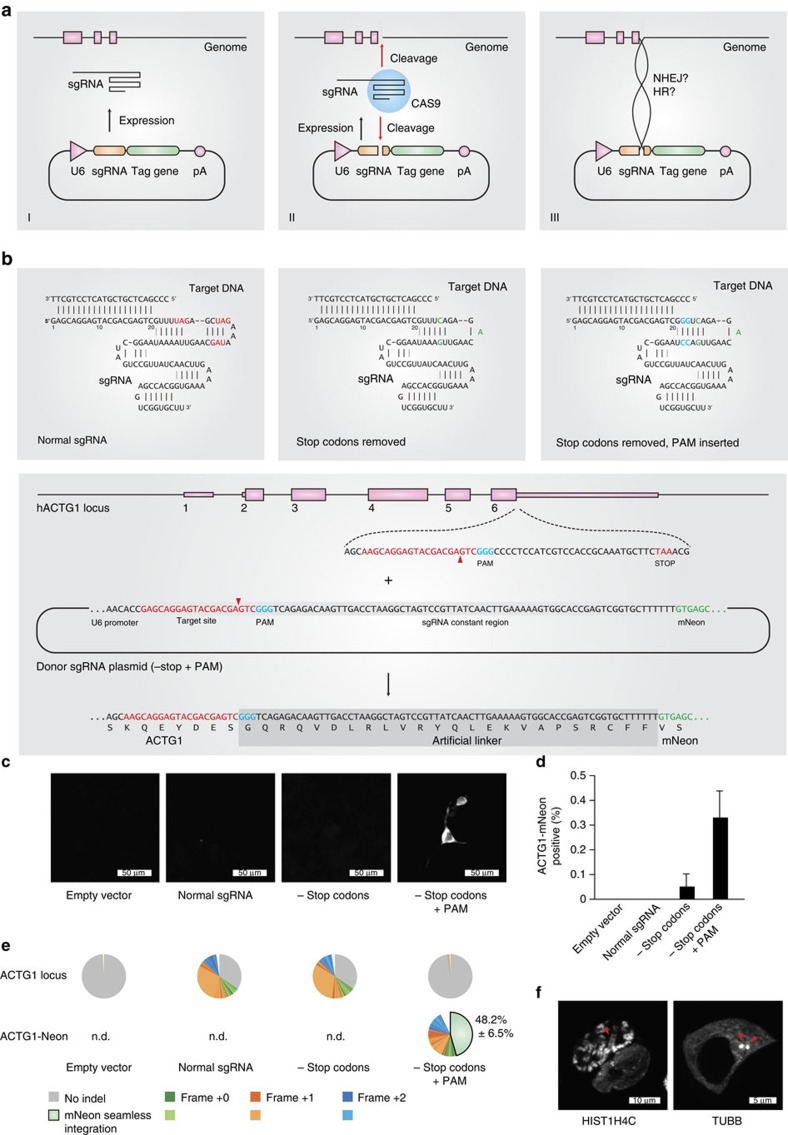
Self-cleaving sgRNA plasmid integration. (**a**) Three steps of a self-cleaving plasmid integration system. I, an sgRNA is expressed from an sgRNA plasmid under the control of a U6 promoter. II, in conjunction with Cas9 protein, the sgRNA cleaves the coding region of a target gene as well as the sgRNA plasmid itself. III, the generated DNA ends of genomic and plasmid DNA are intended to be ligated by the cellular NHEJ or HR machinery, resulting in the genomic integration of the full-length plasmid. (**b**) To allow tag expression after integration of a plasmid according to **a**, stop codons in the constant part of the sgRNA sequence have to be removed. Left panel, normal sgRNA sequence with stop codons in red. Middle panel, stop codons have been removed, base changes in green. Right panel, a PAM motif (blue) has been introduced into the sgRNA sequence to allow efficient cleavage of the sgRNA plasmid. Lower panel, exemplary sequences for tagging the human *ACTG1* gene using a −stop+PAM construct. (**c**) Microscopic images of HEK 293 cells transfected with different integration plasmids. (**d**) Image quantification of *ACTG1*-mNeon-positive cells. Shown are mean values+s.e.m. from three independent biological replicates. (**e**) Deep sequencing analysis of random genomic editing events using primer pairs spanning the targeting region (upper pie charts) and mNeon integration events using primer pairs upstream of the targeting region and within the mNeon gene (lower pie charts). Frame shifts are colour-coded as indicated. The shades of each colour allow to distinguish individual indel events (see legend). Shown are representative results of one out of three independent biological replicates. The percentages indicated are mean values+s.e.m. from three independent biological replicates. ND, not determined. (**f**) Confocal images of HEK 293 cells transfected with targeting constructs for the human *HIST1H4C* and *TUBB* genes. Red arrows indicate an individual chromosome (left panel) or the microtubule-organizing center (right panel).

When generating knockout cell lines using the CRISPR-Cas9 system, we frequently observed that sgRNA expression plasmids or parts thereof can integrate at the respective target sites. We envisioned two possible mechanisms for this phenomenon: either the 20 bp homology of the sgRNA plasmid to the genomic target site led to a single-crossover event as previously described for homologous recombination using long-homology arms^19^,^20^,^21^, or a fraction of non-intact plasmid DNA integrated via mechanisms involving NHEJ (refs 23, 24, 25). In principle, both mechanisms would permit the establishing of a directed gene tagging method based on the use of a plasmid which would encode for a genome-targeting sgRNA and at the same time provide a tag gene to be integrated at the site of the Cas9-induced genomic lesion (Fig. 1a). Considering the possibility of a single-crossover event between the targeting plasmid and the genome occurring at the variable region of the sgRNA, it has to be kept in mind that the integration of the sgRNA sequence at the target region would prevent the creation of a new open reading frame (target gene+tag) as the constant region of the canonical sgRNA encodes for translation termination codons in all reading frames. To avoid this problem, we removed all stop codons from one putative reading frame of the constant region of an sgRNA sequence targeting the last coding exon of the human actin gamma 1 gene (exon 6, *ACTG1*) based on published results from sgRNA mutagenesis studies^30^ (Fig. 1b top panel, red letters). We furthermore generated an additional variant of this sgRNA construct by introducing a PAM motif (NGG) in the constant part of the sgRNA (Fig. 1b top panel, blue letters). This additional feature would allow the linearization of the sgRNA-encoding plasmid and might thereby enhance integration efficiency, should a non-HDR-dependent repair mechanism be at play. In these constructs we positioned the open reading frame of the fluorescent protein mNeon (ref. 31) immediately downstream of the RNA Pol III termination signal of the encoded sgRNA transcript. Should this self-inserting plasmid integrate as intended, the actin gamma 1 protein would be expressed with a C-terminal mNeon tag fused by a 24-amino acid linker sequence that is determined by the constant region of the sgRNA (Fig. 1b, lower panel). Testing these three different constructs in HEK 293 cells stably expressing Cas9 revealed that only the double-modified sgRNA construct (−stop codons/+PAM) yielded cells with a cytosolic pattern of green fluorescence (0.33%; Fig. 1c,d), whereas the other two constructs (+stop codons/−PAM and −stop codons/−PAM) largely failed to produce mNeon fluorescent cells. Using deep sequencing, we observed that the double-modified sgRNA targeting construct resulted in a large proportion of in-frame integration events of the mNeon cassette at the *ACTG1* locus (Fig. 1e, lower panel). The predominant sequence of this novel *ACTG1*-mNeon open reading frame was a seamless fusion of both sequences (48.2%), whereas the remaining fusion events contained mostly deletions that were scattered around the target site (Supplementary Fig. 1a). Analysing the genomic targeting region itself by deep sequencing revealed that the canonical sgRNA construct, as well as the sgRNA devoid of stop codons exerted strong genome editing activity, whereas introduction of the PAM motif into the sgRNA vector reduced the overall genome editing activity (Fig. 1e, upper panel). This is most likely attributable to the fact that self-cleavage of the targeting plasmid by its encoded sgRNA limits sgRNA expression by negative feedback. Nevertheless, despite this major drawback of greatly reduced genome editing activity, applying this methodology to other target loci (for example, *HIST1H4C* and *TUBB*) reliably produced cells with correctly tagged genes with a similar efficiency (0.3–0.4%) as the *ACTG1* targeting approach (Fig. 1f, Supplementary Fig. 1b,c). The requirement of the donor template being processed itself suggested that this tagging approach was independent of HDR, yet rather dependent on NHEJ-mediated integration of the processed plasmid into the site of the DSB.

### Improved gene tagging using a separate donor plasmid

**Figure 2:**
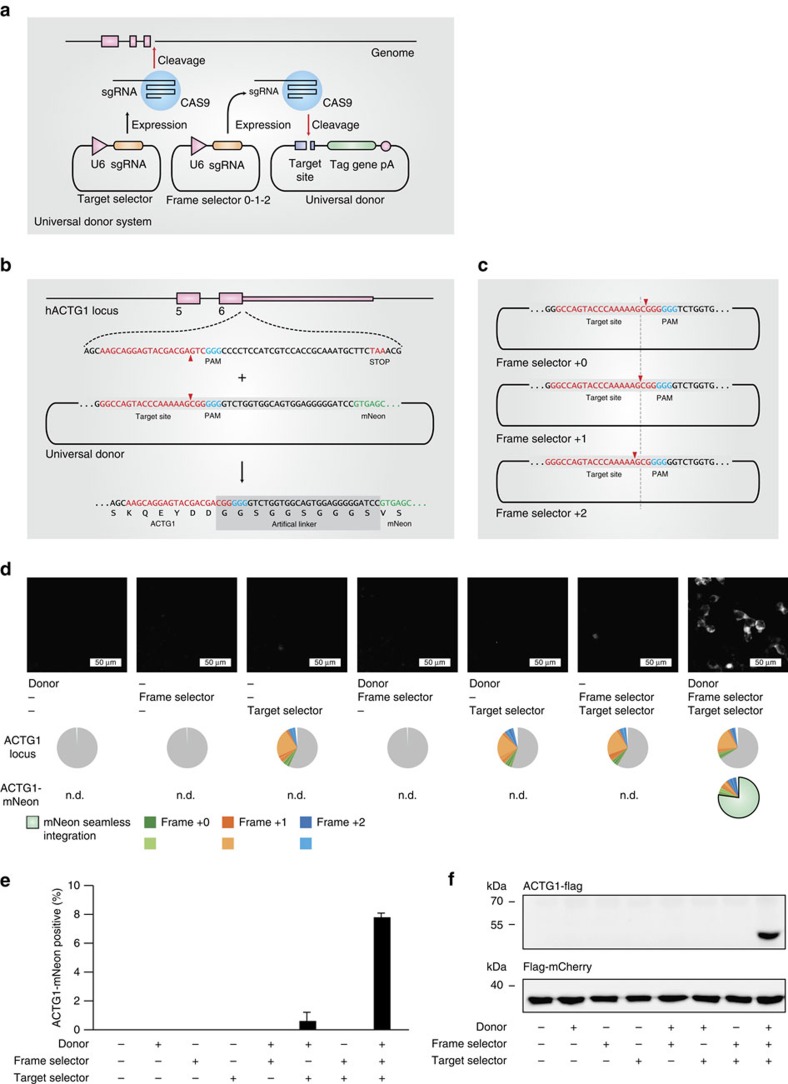
Modular three-plasmid gene tagging system. (**a**) Three-plasmid tagging system. A target selector plasmid expresses an sgRNA targeting a gene of interest. A frame selector plasmid expresses an sgRNA targeting the donor plasmid. A universal donor plasmid contains the tag gene. (**b**) Sequence details of the universal donor plasmid when integrating into the human *ACTG1* gene. (**c**) Due to a poly-G stretch within the target site of the universal donor plasmid it can be cleaved at three adjacent nucleotide positions, which allows specifying the frame of integration at the time of transfection. (**d**) Fluorescence imaging and deep sequencing analysis of *ACTG1*-mNeon gene tagging using a three-plasmid system and different plasmid combinations. ND, not determined. (**e**) Image quantification of *ACTG1*-mNeon-positive cells. Shown are mean values+s.e.m. from three independent biological replicates. (**f**) Immunoblotting of *ACTG1*-Flag gene tagging using a three-plasmid system and different plasmid combinations.

In addition to the low efficiency, the here-described system still bears three limitations: first, only few Cas9 target sites in a given gene are available for tagging, since a specific reading frame must be targeted due to stop codons in the constant part of the sgRNA when translated in other reading frames. This considerably limits the number of targeting possibilities, which is especially of concern for a restricted targeting window (for example, tagging of the last coding exon for C-terminal fusion tagging of a protein). Second, the integrated linker amino acids are dictated by the sgRNA sequence and thus are likely inferior to a generic linker peptide. Third, a functional U6 promoter is integrated into the locus of a tagged gene, which might interfere with physiological gene expression. To overcome these limitations, we devised a second version of the tagging system, based on the combination of three plasmids (Fig. 2a): Here, a target selector expresses a canonical sgRNA cleaving the target gene, a frame selector expresses an sgRNA cleaving a donor plasmid, and the universal donor plasmid contains a Cas9 target site upstream of an encoded short, unstructured linker peptide (GGSGGSGGGS) in frame with the mNeon tag gene (Fig. 2b). Due to its design, the donor can be cleaved at three adjacent nucleotide positions by selecting the frame selector sgRNA accordingly, which allows choosing the correct reading frame for the gene to be tagged (Fig. 2c). With this system, any site in a coding region can be targeted regardless of the reading frame, no U6 promoter is integrated into the targeted genomic locus and the translated protein will be fused to the tag by a short linker peptide. When transfecting all combinations of the three plasmids into HEK 293-Cas9 cells, only the complete set resulted in a considerable number of mNeon-positive cells (Fig. 2d,e), with only minor fluorescence signal detected without a frame selector. Deep sequencing confirmed that on-target genome editing was solely dependent on the target selector plasmid (Fig. 2d, middle panel). Using the same method, we also integrated a FLAG epitope tag into the *ACTG1* gene, which was readily detected by western blot when the complete set of plasmids was transfected (Fig. 2f). At the same time, efficient gene tagging was also achieved in the difficult-to-transfect cell line THP1 using co-electroporation of the plasmids and subsequent fluorescence-activated cell sorting (Supplementary Fig. 2a,b).

### Fidelity assessment of reading-frame selection

**Figure 3:**
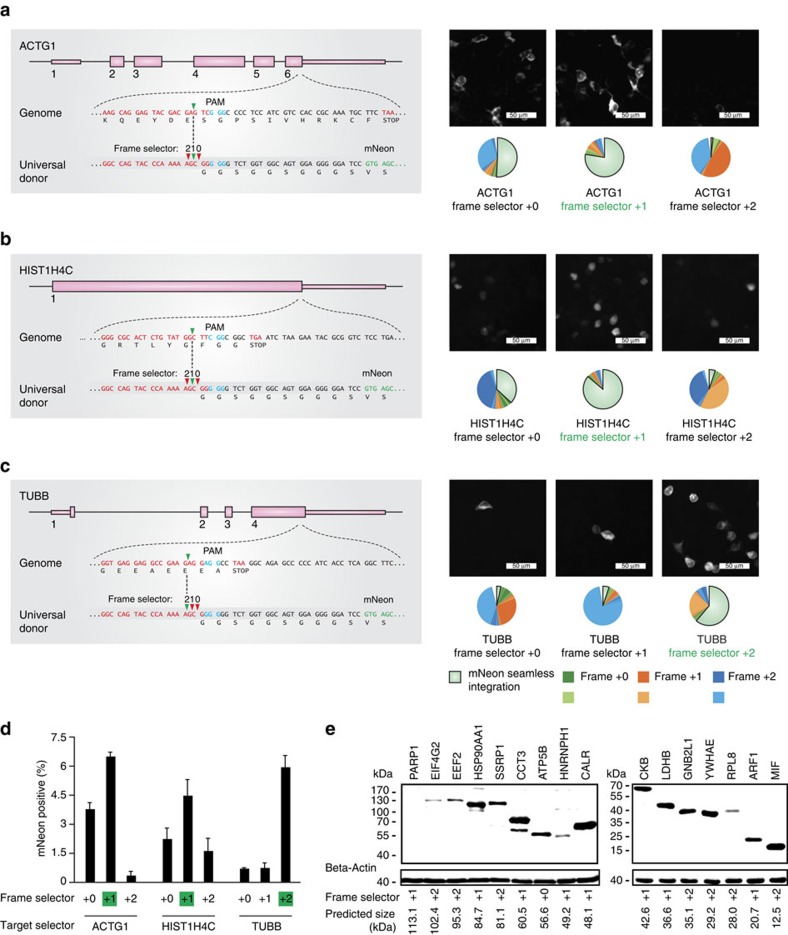
Flexible frame selection of CRISPaint gene tagging. (**a–c**, left panels) Scheme of the exon structure of three genes targeted by CRISPaint. (**a–c**, right panels) Fluorescence imaging and deep sequencing analysis of *ACTG1*-mNeon (**a**) *HIST1H4C*-mNeon (**b**) and *TUBB*-mNeon (**c**) gene tagging using different frame selector plasmids. The frame selector predicted for in-frame tagging is marked in green. (**d**) Image quantification of mNeon tagging-positive cells. Shown are mean values+s.e.m. from three independent biological replicates. (**e**) Immunoblotting result of CRISPaint-mediated tagging of 16 human genes with a 3 × FLAG tag. Indicated below are the predicted protein sizes and frame selectors used.

To verify the frame selection feature of our approach, we targeted three genes (*ACTG1*, *HIST1H4C* and *TUBB*) using all three possible combinations of frame selector plasmids (Fig. 3a–c). To avoid the possibility of re-cutting of the newly generated fusion site by the target selector sgRNA, we avoided target regions in which the three nucleotides upstream of the PAM region would resemble the donor target site (Supplementary Note 1 and Supplementary Fig. 3). While tagging of the *ACTG1* and *HIST1H4C* genes was most efficient using frame selector +1 (Fig. 3a,b,d), tagging of the *TUBB* gene was most efficient when using frame selector +2 (Fig. 3c,d). These results are well in line with the positioning of the sgRNA target sites in the coding region of the targeted genes. Furthermore, deep sequencing of the integration events confirmed a flexible frame selectivity, with 61.5–86.7% seamless fusion achieved using the correct frame selector. Comparing the three frame selectors irrespective of the intended in-frame fusion event revealed that frame selector +1 was superior to frame selectors +0 and +2 (Fig. 3a–c). At closer investigation, we observed that especially frame selector +0 gave rise also to integration events bearing a +1 offset relative to a seamless integration. Indeed, we found that this +1 frame shift was predominantly caused by the duplication of the base at position -4 of the donor plasmid with respect to the PAM motif (Supplementary Fig. 4, Supplementary Note 2). When considering single base insertions only, we observed a highly negative correlation with the overall integration fidelity (Supplementary Fig. 5a), which suggests that this phenomenon largely accounts for frame selector-dependent variability of integration fidelity (Supplementary Fig. 5b). However, by tagging 16 independent genes with a FLAG tag using all three frame selectors, we found that 15 out of 16 encoded proteins were detectable at approximately the predicted size on an immunoblot (Fig. 3e). Of note, in some cases the detected protein sizes were larger than predicted (for example, CALR), most likely attributable to post-translational modification events.

### Enhancement of tagging frequencies using puromycin selection

**Figure 4:**
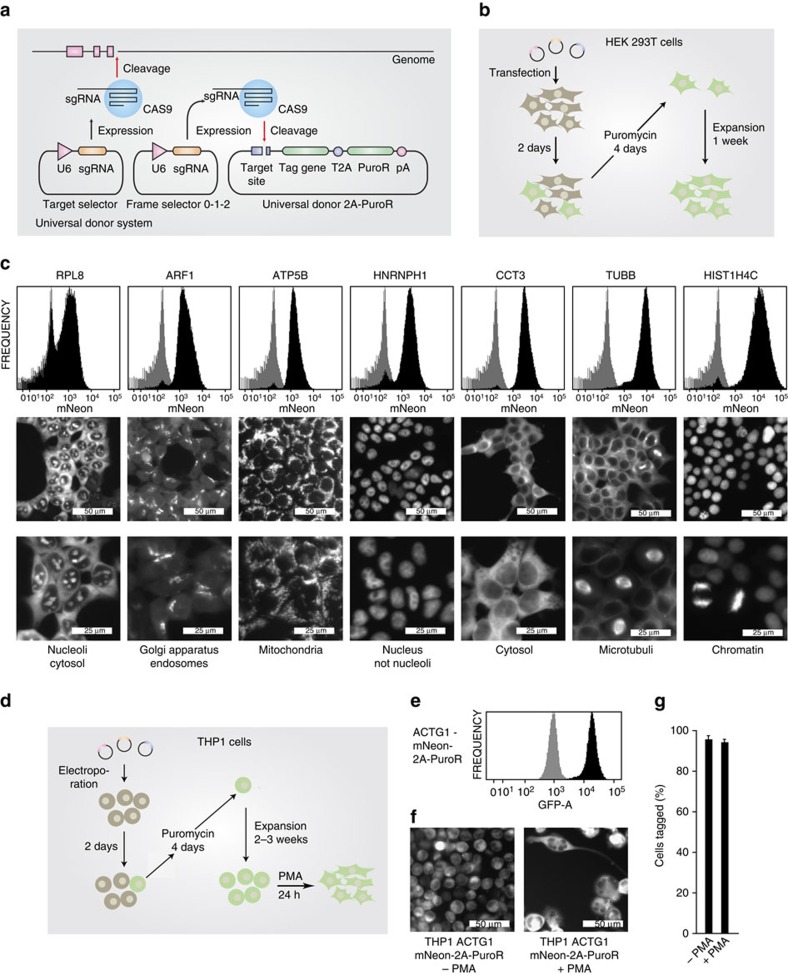
Efficient enrichment of in-frame tagged cells by antibiotic selection. (**a**) Scheme of a three-plasmid tagging system that allows selection for positively tagged cells by expressing a puromycin resistance gene separated from the tag gene by a T2A peptide. (**b**) Experimental set-up and timeline for selection-based gene tagging in HEK 293T cells. (**c**) The endogenous gene loci of seven genes were C-terminally tagged in HEK 293T cells according to the strategy outlined in **b**. After selection, cells were analysed for mNeon fluorescence by fluorescence-activated cell sorting (FACS) and data are depicted as histogram plots. Tagged cells are shown in black, whereas a reference histogram of mock-treated cells is depicted in grey. In addition, cells were subjected to fluorescence imaging of tag gene expression. (**d**) Experimental set-up and timeline for selection-based gene tagging in the difficult-to-transfect cell line THP1. (**e**) FACS-based assessment of mNeon fusion-gene expression in THP1 cells after tagging of the endogenous *ACTG1* gene and subsequent puromycin selection according to **d**. (**f**) Fluorescence imaging of tag gene expression after selection according to **d** and after additional PMA treatment to induce differentiation. (**g**) Image quantification of mNeon tagging-positive cells generated according to **f**. Shown are mean values+s.e.m. from two independent visual fields. Results are representative for two independent experiments.

With the current set-up, we routinely achieved in-frame tagging efficiencies of 4–8% without further enrichment. Taking into account that the donor construct integrates into the target DSB with random orientation and that not all integration events are in frame with the gene of interest, a tagging frequency of 4–8% suggests a very high integration efficiency that approaches ∼50–75% of the DSB-induction frequency at the target site itself (compare Fig. 2d,e). Nevertheless, to further increase the proportion of correctly tagged cells, we introduced a sequence encoding for a self-cleaving T2A peptide followed by a puromycin resistance gene (puromycin *N*-acetyl-transferase) downstream the tag. In this configuration, the protein of interest is expressed with a C-terminal tag in frame with puromycin *N*-acetyl-transferase and application of puromycin should deplete cells in which donor constructs have integrated in the wrong orientation or reading frame. Using this novel donor system (universal donor 2A-Puro), we targeted seven genes with mNeon in HEK 293T cells using the appropriate frame selectors. Two days following transfection, we applied selection pressure using puromycin for 4 days and then expanded the cells for one additional week (Fig. 4b). While gene tagging efficiencies without antibiotic selection were in the same range as previously observed, puromycin-selected cultures contained largely uniform tag-positive cells (Fig. 4c). The intracellular localization of all tagged proteins recapitulated known protein localizations, allowing the visualization of cellular organelles such as mitochondria or nuclei (Fig. 4c). Similar tagging efficiencies were obtained when *ACTG1* was targeted in THP1 cells with the mNeon universal donor 2A-Puro construct (Fig. 4d–g). Here a frequency of 95.7% tag-positive cells was obtained following 4 days of selection pressure with puromycin and an additional expansion period of 3 weeks.

### Generation of a minimal donor plasmid

**Figure 5:**
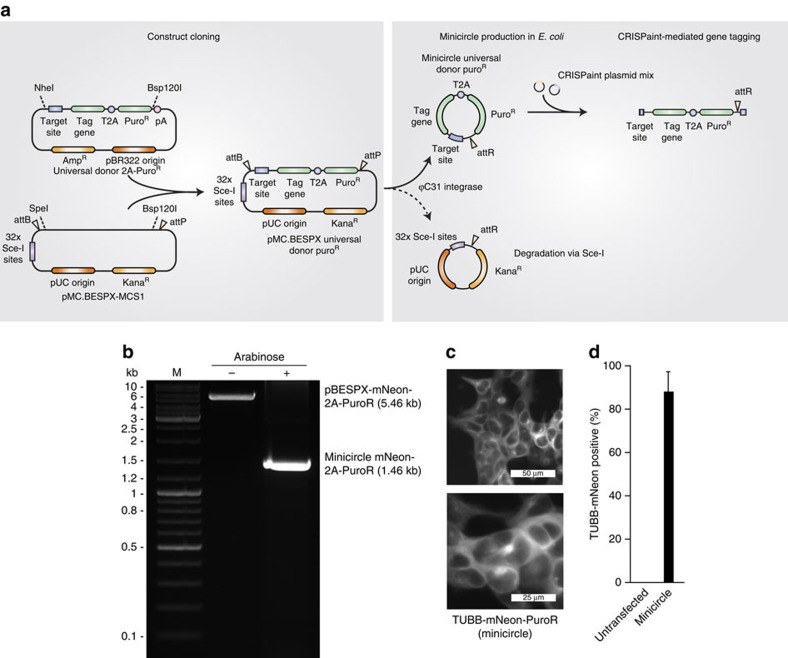
Minicircle DNA-based insertion tagging. (**a**) Inserts of the universal donor 2A-PuroR construct library are subcloned into pMC.BESPX-MCS1 via NheI or SpeI and Bsp120I. The resulting vector can be used to generate a minicircle universal donor construct in an *E. coli* strain that expresses inducible ϕC31 integrase and I-SceI endonuclease. The resulting minicircle DNA contains the universal donor PuroR cassette and is devoid of bacterial plasmid backbone sequences. On delivery of the minicircle donor construct in conjunction with the CRISPaint plasmid mix, the donor plasmid is cut and integrated into the DSB at the genomic target region. (**b**) Agarose gel confirming the elimination of plasmid backbone sequences from donor DNA by cultivation of transformed *E. coli* strain ZYCY10P3S2T with arabinose induction solution for 5 hours before DNA preparation. DNA was linearized with BamHI before loading on the gel. (**c**) Fluorescence microscopy of HEK 293T cells with an mNeon-2A-PuroR-tagged *TUBB* gene using minicircle DNA as a donor and selected with puromycin for 4 days. Shown is a representative result from two biological replicates. (**d**) Quantification of *TUBB*-mNeon-positive cells using minicircle DNA as a donor and selected with puromycin for four days. Shown are mean values+s.e.m. from two independent biological replicates.

To eliminate the need of integrating bacterial backbone sequences into endogenous genomic loci, which might interfere with expression levels or genomic integrity, we generated the donor DNA as minicircle DNA using a bacterial strain that allows recombination of donor plasmids (Fig. 5a,b). After tagging the *TUBB* gene with mNeon using minicircle donor DNA, we observed similar tagging efficiencies as with plasmid donor DNA (Fig. 5c,d).

Due to the requirement of a PAM motif next to the termination codon of the target gene, C-terminal amino acids can be lost from the encoded protein. To solve this possible limitation, a modified donor DNA plasmid was designed that allows to re-introduce amino acids that are lost due to restrictions of target site selection (Supplementary Fig. 6a), thereby allowing scarless gene tagging using CRISPaint. We successfully applied this scarless approach to tag the endogenous *ACTG1* gene with mNeon (Supplementary Fig. 6b), confirming high integration fidelity using deep sequencing (Supplementary Fig. 6c).

### Characterization of the mechanism of CRISPaint gene tagging

**Figure 6:**
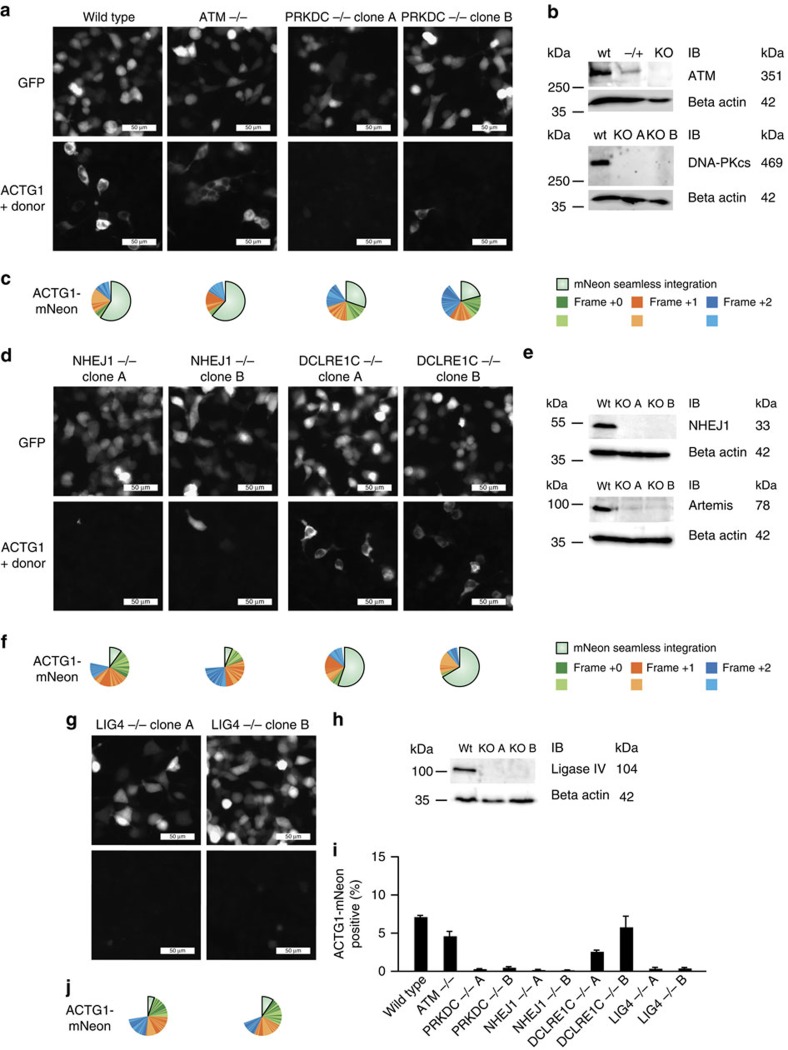
Involvement of cNHEJ repair components in CRISPaint-mediated gene tagging. (**a**,**d**,**g**) Fluorescence imaging of GFP expression from a control plasmid (upper panels) or *ACTG1*-mNeon gene tagging (lower panels) in indicated gene-deficient cell lines. (**b**,**e**,**h**) Immunoblot validation of CRISPR-Cas9 generated knockout cell lines that were pre-validated by deep sequencing to bear all-allelic frame shift mutations (knockout (KO) A, B) or heterozygous mutations (−/+). Size marker bands are indicated in the first column; expected protein sizes are given in the last column. (**c**,**f**,**j**) Deep sequencing analysis of the fusion junctions created by NHEJ-mediated gene tagging. (**i**) Image quantification of *ACTG1*-mNeon-positive cells. Shown are mean values+s.e.m. from three independent biological replicates.

Finally, we strived to characterize the cellular DSB repair machinery components responsible for CRISPaint to work. Using CRISPR-Cas9, we generated HEK 293-Cas9 cell clones deficient for the DNA damage repair-associated genes *ATM*, *PRKDC* (DNA-PKcs), *NHEJ1* (XLF), *DCLRE1C* (Artemis), and *LIG4* (Ligase-4) and validated them by deep sequencing (Supplementary Fig. 7) and immunoblotting (Fig. 6b,e,h). All cell clones displayed normal cell morphology and could be readily transfected with a green fluorescent protein (GFP) expression plasmid (Fig. 6a,d,g, upper panels). However, when assessing CRISPaint efficiency in these cells by tagging *ACTG1* with mNeon, we found that the cNHEJ components DNA-PKcs, XLF and Ligase-4 were essential for efficient gene tagging, whereas Artemis and ATM were dispensable (Fig. 6).

## Discussion

We here describe a three component CRISPR-assisted insertion tagging (CRISPaint) method, which constitutes a simple, efficient and versatile approach to tag an endogenous gene of interest. Applying this system only requires the construction of a single sgRNA vector that targets the genomic region of choice, while a second plasmid used to specify the reading frame of the insertion as well as a third universal donor plasmid containing the tag itself can be picked from a pre-made library. Tag-expressing cells can already be detected 1–2 days following plasmid delivery by transfection or electroporation. In easy to transfect cell lines, no enrichment strategies, such as the selection for a heterologous antibiotic resistance, are required to obtain a considerable percentage of CRISPR-modified cells. Nevertheless, an even higher proportion of uniformly tagged cells can be achieved when using a puromycin resistance cassette, enabling gene tagging also in cells that are difficult to transfect.

Next to the donor constructs used here, we also provide plasmids for tagging with Luciferase (T2A-Gaussia Luciferase, NanoLuc), various fluorescent proteins (TagGFP2, TagBFP, TagRFP and T2A-TurboGFP-PEST), small epitope tags for sensitive detection or affinity purification approaches (HA, Myc, Strep tag II, Streptavidin-binding peptide, AviTag, SpyTag, SunTag v4), a controllable destabilization tag (ProtoTuner DD), and tags to assess protein–protein interaction (split-TEV protease). All these tags are available in the universal donor format with or without the puromycin resistance cassette (Supplementary Data 1). Moreover, to simplify the application of the method for C-terminal tagging approaches, we supply a list of pre-selected, sequence-optimized target sites (Supplementary Note 1) close to translation termination codons of nearly all human and mouse protein coding genes in conjunction with the required frame selector (Supplementary Data 2 and 3). Of note, we found that only 5.4 amino acids are lost on average on C-terminal protein tagging of human genes using CRISPaint.

While primarily designed to obtain C-terminally tagged endogenous proteins, this approach could also be used to introduce any type of heterologous genetic material at any site within the genome. Possible applications could be the insertion of gene trapping cassettes into intronic regions of transcribed genes, the specific insertion of genetic variants of interest into exonic regions or the insertion of expression cassettes into ‘safe harbour regions'. Apart from that, the CRISPaint approach could also be useful to express proteins from endogenous promoters in a constant, physiological and predictable fashion by appending a transgene to endogenous genes with a T2A peptide linker. Of note, the seven target genes studied here already offer a broad variety of defined expression levels (Fig. 4c) that could readily be exploited for controlled transgene expression.

It is important to note that the CRISPaint donor constructs integrate into the target loci in random orientation. Of note, however, the insertion frequencies given in this study were assessed by quantitative microscopy so that only integration events in the correct orientation and reading frame were detected. In practical terms, any sequencing-based genotyping method will readily discriminate between the two orientations of donor construct integration.

The major advantage of CRISPaint over HDR-directed introduction of heterologous genetic material using a donor template is its simplicity and modularity at high efficiency. The fact that no site-specific donor template with homology arms has to be generated greatly enhances the overall time frame to obtain genetically tagged cells. Moreover, applying this system, we regularly achieved site-specific, seamless integration efficiencies within the range of 4–8% in HEK cells without further selection, a frequency that lies within the same range as optimized HDR-dependent integration strategies^32^.

Apart from that, CRISPaint epitope tagging should greatly alleviate the requirement for protein-specific antibodies for endogenous gene expression quantification, protein localization or immuno-precipitation studies. Of note, using established epitope-specific antibodies in conjunction with calibration peptides will even allow absolute protein quantification. Despite many advantages, it should be noted that HDR-dependent integration of heterologous DNA cassettes will still be the method of choice if a very specific alteration of a genetic locus is desired (for example, introduction of SNVs) or if no appropriate target site can be found in proximity to the locus of interest.

Apart from the minicircle donor, the CRISPaint universal donor vectors harbour a strong poly A signal downstream of the tag gene of interest. This means that the endogenous 3′ UTR, which can regulate gene expression at the posttranscriptional level, is not included in the resulting messenger RNA. Moreover, if the donor plasmid backbone is integrated downstream of the target locus, it could evoke epigenetic modifications and thus alteration of the physiological gene expression level. To circumvent these limitations, the universal donor plasmid can be prepared as a minicircle DNA, in which the bacterial plasmid backbone is removed before delivery into the target cells. We implemented this option in all constructs of the universal donor library by introducing restriction sites compatible with pMC.BESPX-MCS1, a recently described backbone for highly efficient minicircle DNA production^33^. Moreover, the same recombination sites used for minicircle DNA preparation could also be used to remove the plasmid backbone following integration into eukaryotic genomes using transient expression of ϕC31 integrase^34^.

In this study, we provide a comprehensive genetic dissection of the contribution of different repair pathways to CRISPR-Cas9-mediated genome integration events in human cells, exceeding the depth of prior studies^35^,^36^,^37^. Our results demonstrate that cNHEJ is required to direct the integration of DNA cassettes into CRISPR-Cas9-mediated DSBs. As such, the presence of DNA-PKcs, XLF and Lig-4 was critically required for producing mNeon-expressing cells using CRISPaint. Fusion events harbouring deletions at the junction of the genomic target region and the donor sequence suggestive of aNHEJ were also detected, but only made up a minor fraction of events. Moreover, if present, these microhomology-associated mutations were scattered across the fusion region with variable deletion sizes. As such, considering their low frequency and high variability, we understand that microhomology-associated integration events are far less projectable than cNHEJ-mediated insertions dominantly obtained by CRISPaint. Interestingly, in some targeting experiments we observed the presence of a second prominent fusion event besides the expected primary fusion sequence. On closer analysis, this phenomenon could be attributed to a single-nucleotide duplication located four bases upstream of the PAM sequence of the donor plasmid, that is, adjacent to the putative Cas9 cut site. Additional studies will be required to determine whether this phenomenon might be depending on the base composition of the target site, which would allow further rational enhancement of the CRISPaint system.

Collectively, CRISPaint provides a simple, precise and rapid methodology to integrate heterologous genetic material via cNHEJ in CRISPR-Cas9-accessible cellular systems. Given its high degree of modularity, CRISPaint not only allows the conduction of single-gene targeting experiments, but it will also enable large scale, site-directed gene insertion studies.

## Methods

### Plasmid cloning

sgRNA expression plasmids were assembled using LIC (ref. 38). Tag genes were synthesized as gBlocks (IDT). The scarless donor plasmid for *ACTG1* was assembled by annealing two oligonucleotides (5′-CTAGCGGGCCAGTACCCAAAAAAAGCGGCCCCTCCATCGTCCACCGCAAATGCTTCGGGTCTGGTGGCAGTGGAGGGG-3′, 5′-GATCCCCCTCCACTGCCACCAGACCCGAAGCATTTGCGGTGGACGATGGAGGGGCCGCTTTTTTTGGGTACTGGCCCG-3′) and subsequent ligation into an mNeon-donor plasmid linearized by NheI and BamHI. A general approach to generate scarless donor constructs is provided in Supplementary Note 3. All plasmid sequences are listed in Supplementary Data 1 and 4.

### Plasmid transfection

Throughout the study HEK blue-mCherry-CAS9 cells (HEK 293 cells) or HEK 293T cells were used^38^. Cells were plated at a density of 3 × 10^4^ cells per 96-well. On the next day, plasmids were transfected using GeneJuice transfection reagent (Merk Millipore) according to manufacturer's protocol. In a typical tagging experiment, 50 ng target selector plasmid, 50 ng frame selector plasmid and 100 ng donor plasmid were co-transfected per 96-well.

### Plasmid electroporation

THP-1 cells were diluted to a density of 2 × 10^5^ cells per ml and grown over night. The next day, 2.5 × 10^6^ cells were mixed with 1.25 μg frame selector plasmid, 1.25 μg target selector plasmid and 2.5 μg donor plasmid in 250 μl Opti-MEM and were transferred to a 4 mm cuvette. Electroporation was performed using a Gene Pulser electroporator (Bio-Rad Laboratories) with the settings: exponential pulse, 250 V, 950 μF.

### Puromycin selection

Two days after transfection, the growth medium was supplemented with 3 μg per ml puromycin. Four days later, cells were washed once and were allowed to recover in puromycin-free medium for up to 2 weeks.

### Flow cytometry

Cells were trypsinized if necessary and analysed using a BD LSR-II flow cytometer using standard settings for measuring GFP fluorescence.

### Microscopy

Two days after transfection, epifluorescence images were acquired using a Zeiss Observer.Z1 inverted microscope equipped with a × 20 Plan-Neofluar objective or a Zeiss Axio Vert.A1 inverted microscope equipped with a × 40 Plan-Neofluar objective. Confocal microscopy was performed on a Leica SP5 SMD confocal microscope with a × 63 water-immersion objective.

### Image quantification

About 200 total cells per image and the fraction of mNeon-positive cells were counted using ImageJ/FIJI and the Cell Counter plugin.

### Genomic DNA preparation

The medium was removed and the cells were lysed in 30 μl of the following lysis buffer: 0.2 mg per ml proteinase K, 1 mM CaCl_2_, 3 mM MgCl_2_, 1 mM EDTA, 1% Triton X 100 and 10 mM Tris pH 7.5. The reactions were incubated at 65 °C for 10 min and at 95 °C for 15 min.

### Dual PCR barcoding

First-level PCR reactions are performed using 4 μl PCR-compatible lysate as a template for a 25 μl Phusion (Thermo Scientific) PCR reaction according to the manufacturer's protocol (annealing temperature: 60 °C, elongation time: 15 s, 19 cycles). Of this reaction, 4 μl are transferred to a second-level PCR using the same cycling conditions and a second set of primers. For all primer sequences see Supplementary Data 5.

### Deep sequencing

Crude PCR products were pooled and size-separated using a 1.5 % agarose gel run at 100 V. After visualization with ethidium bromide under UV light, DNA bands from 350 to 450 bp were cut out and purified using Jena Analytik innuPrep gel extraction kit according to the manufacturer's protocol. Eluted DNA was precipitated by adding 0.1 volumes of 3 M NaOAc (pH 5.2) and 1.1 volumes of isopropanol. After centrifugation for 15 min at 4 °C, the resulting pellets were washed once in 70% EtOH and air-dried. 30 μl water was added, non-soluble fractions were spun down and removed, and the DNA concentration was quantified using a NanoDrop spectrophotometer system. DNA was diluted and deep sequencing was performed according to the manufacturer's protocol using the MiSeq (Illumina) bench top sequencing system. Data were obtained in FASTQ format.

### Deep sequencing data evaluation

Sequencing data was evaluated using the web tool OutKnocker.org (ref. 39) using standard parameters. NHEJ-mediated mutagenesis frequencies and NHEJ-mediated tag integration fidelities were analysed by separate experiments using either two locus-specific PCR primers, or one locus- and one tag-specific PCR primer, respectively. Of note, these results cannot be compared quantitatively and thus do not replace quantitative microscopy.

### Immunoblots

Cells were lysed in 50 μl of Laemmli buffer per 96-well, heated to 95 °C for 10 min and run on 8–12% SDS-polyacrylamide gel electrophoresis gels at 100 V. After blotting to nitrocellulose membranes and blocking in 5% milk, flag-tagged proteins were visualized by Horseradish peroxidase-conjugated ANTI-FLAG-M2 antibody (Sigma A8592), while for knockout validation the following primary antibodies were used: Artemis D7O8V rabbit mAb (Cell Signaling 13381); NHEJ1 XLF rabbit Ab (Cell Signaling 2854); DNA Ligase IV D5N5N rabbit mAb (Cell Signaling 14649); DNA-PK rabbit Ab (Cell Signaling 4602); and ATM 2C1 mouse mAb (Abcam ab78). All primary antibodies were used at a dilution of 1:1000. Supplementary Fig. 8 displays uncropped immunoblots of Fig. 6.

### CRISPR-Cas9 gene targeting

Knockout cell lines were generated by transfecting gRNA expression plasmids into HEK blue-Cas9 cells, targeting the coding regions of the genes *ATM* (5′-GGAGAGAGCCAAAGTACCATAGG-3′), *LIG4* (5′-AGATATTGAGCACATTGAGAAGG-3′), *PRKDC* (5′-GCCGGTCATCAACTGATCCGCGG-3′), *NHEJ1* (5′-GCAAGCTGTAGCCACGCCCATGG-3′) or *DCLRE1C* (5′-GGAGACTTCAGATTGGCGCAAGG-3′). Two days after transfection, single-cell cloning was performed by limiting dilution. After 2 weeks of expansion, clones were genotyped by PCR-based deep sequencing using the Outknocker.org software^39^.

### Cell lines

HEK 293T cells were obtained from Dr E. Latz. HEK blue cells were obtained from Invivogen. THP1 cells were obtained from Dr D. Golenbock. In our lab parental cell lines are routinely tested for the absence of mycoplasma contamination.

### Construct availability

All CRISPaint plasmid constructs, except the ones encoding for mNeon, are available through Addgene.org.

### Data availability

Raw deep sequencing data is made available on SRA (BioProject PRJNA276500 BioSample SAMN03375753). All other data are available from the authors on request.

## Additional information

**Accession codes:** raw deep sequencing data is made available on SRA (BioProject PRJNA276500 BioSample SAMN03375753).

**How to cite this article:** Schmid-Burgk, J.L. *et al*. CRISPaint allows modular base-specific gene tagging using a ligase-4-dependent mechanism. *Nat. Commun.* 7:12338 doi: 10.1038/ncomms12338 (2016).

## Supplementary Material

Supplementary Information Supplementary Figures 1-8 and Supplementary Notes 1-3

Supplementary Data 1 Available CRISPaint tagging plasmids. For each donor available, the table provides the tag name, the linker amino acid sequence, the tag amino acid sequence, the special property of the tag, and possible applications of the tag. Also, the table includes the full sequences of all donor plasmids and information about their availability through Addgene.org.

Supplementary Data 2 Human in-silico library of CRISPR target sites designed for CRISPaint gene tagging. For each targetable human gene, the table provides the official gene symbol the optimal target site, the frame selector that should yield the highest number of tagging-positive cells, the number of bases of the original open reading frame (ORF) that would be lost during CRISPaint gene tagging, as well as the number of amino acids of the wild type protein sequence that would be lost du ring CRISPaint gene tagging.

Supplementary Data 3 Mouse in-silico library of CRISPR target sites designed for CRISPaint gene tagging. For each targetable mouse gene, the table provides the official gene symbol, the optimal target site, the frame selector that should yield the highest number of tagging-positive cells, the number of bases of the original open reading frame (ORF) that would be lost during CRISPaint gene tagging, as well as the number of amino acids of the wild type protein sequence that would be lost during CRISPaint gene tagging.

Supplementary Data 4 Full-length sequences of tagging plasmids.For each construct, the table provides the construct's name, its use, its sequence, and information about its availability through Addgene.org.

Supplementary Data 5 Primer sequences.For each primer, the table provides the amplicon for which the primer has been used, the primer name and the primer sequence. The amplicon is either a gene locus (e.g.*ACTG1* locus) or a tag fusion sequence (e.g. *ACTG1*-mNeon).
