# Supplementary material for: CRISPaint allows modular base-specific gene tagging using a ligase-4-dependent mechanism
Source: Nat Commun. 2016 Jul 28;7:12338. doi: 10.1038/ncomms12338 (PMC4974478; doi:10.1038/ncomms12338)
Supplement: Supplementary Information — Supplementary Figures 1-8 and Supplementary Notes 1-3 [file ncomms12338-s1.pdf]

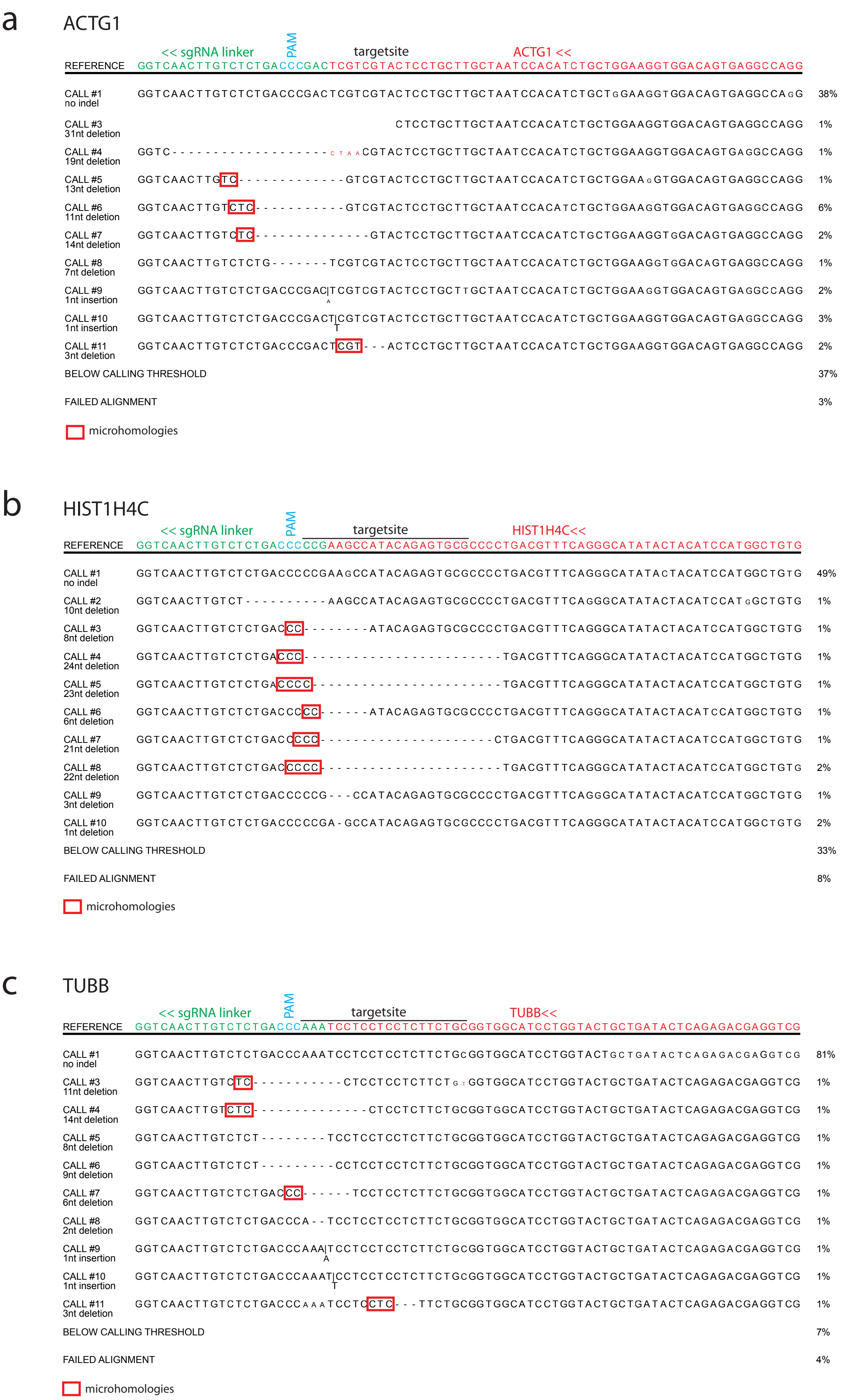

Supplementary Figure 1 | Raw sequencing results from gene tagging experiments using a self-integrating donor. (a-c) Shown are the ten most abundant indel classes in sequenced PCR-amplified genome-donor junctions after mNeon-tagging of **(a)** ACTG1, **(b)** HIST1H4C, and **(c)** TUBB in HEK 293 cells. The seamless fusion sequence is given as a reference. Green letters indicate sequence portions of the sgRNA, blue letters indicate the PAM sequence, red letters indicate genomic sequence portions, and red boxes indicate the presence of microhomologies flanking specific deletions. The data shown results from a single representative experiment out of three, which in the case of (a) is also analyzed in Fig. 1.

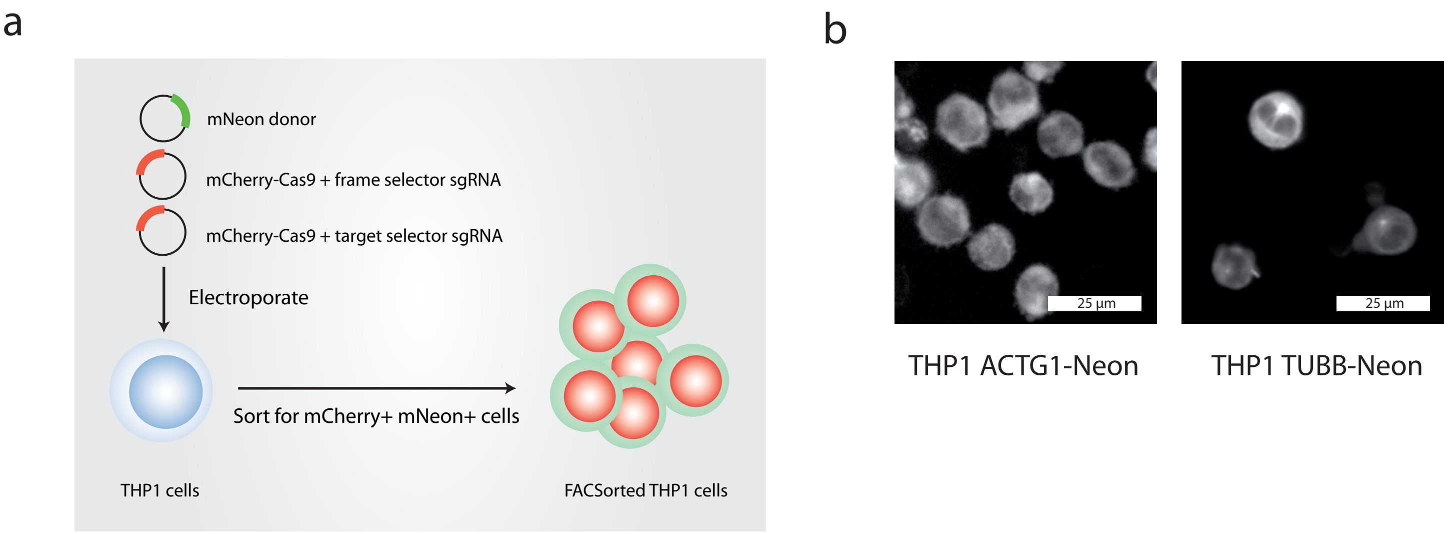

Supplementary Figure 2 | Gene tagging in human myeloid THP1 cells. (a) Outline of the generation of gene-tagged human THP1 cells. First, cells are co-electroporated with the three plasmids indicated. Green and red marks indicate the presence of an mNeon or mCherry coding sequence on the plasmids, respectively. FACS sorting was performed in order to enrich mCherry-mNeon double-positive cells. **(b)** Epifluorescent microscopy of resulting THP1 cells with mNeon-tagged ACTG1 or TUBB genes.

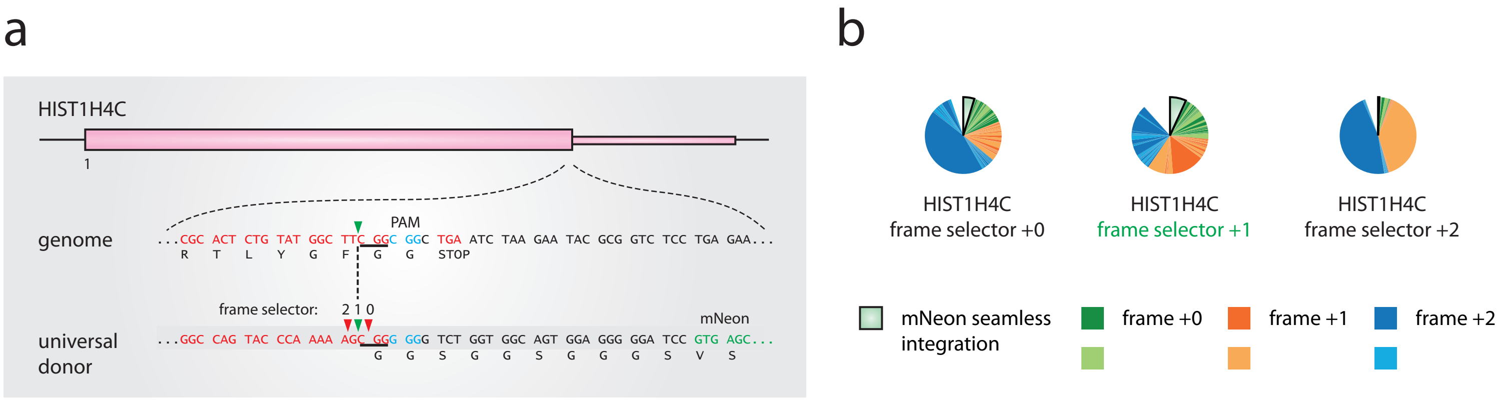

Supplementary Figure 3 | Gene tagging of HIST1H4C using a non-optimal target sgRNA. (a) Scheme of the exon structure of the HIST1H4C gene targeted by CRISPaint using a target site with 3 PAM-adjacent bases identical to the donor target site (identical bases are underlined). **(b)** Deep sequencing analysis of HIST1H4C-mNeon gene tagging events using different frame selector plasmids. The frame selector predicted for in-frame tagging is marked in green.

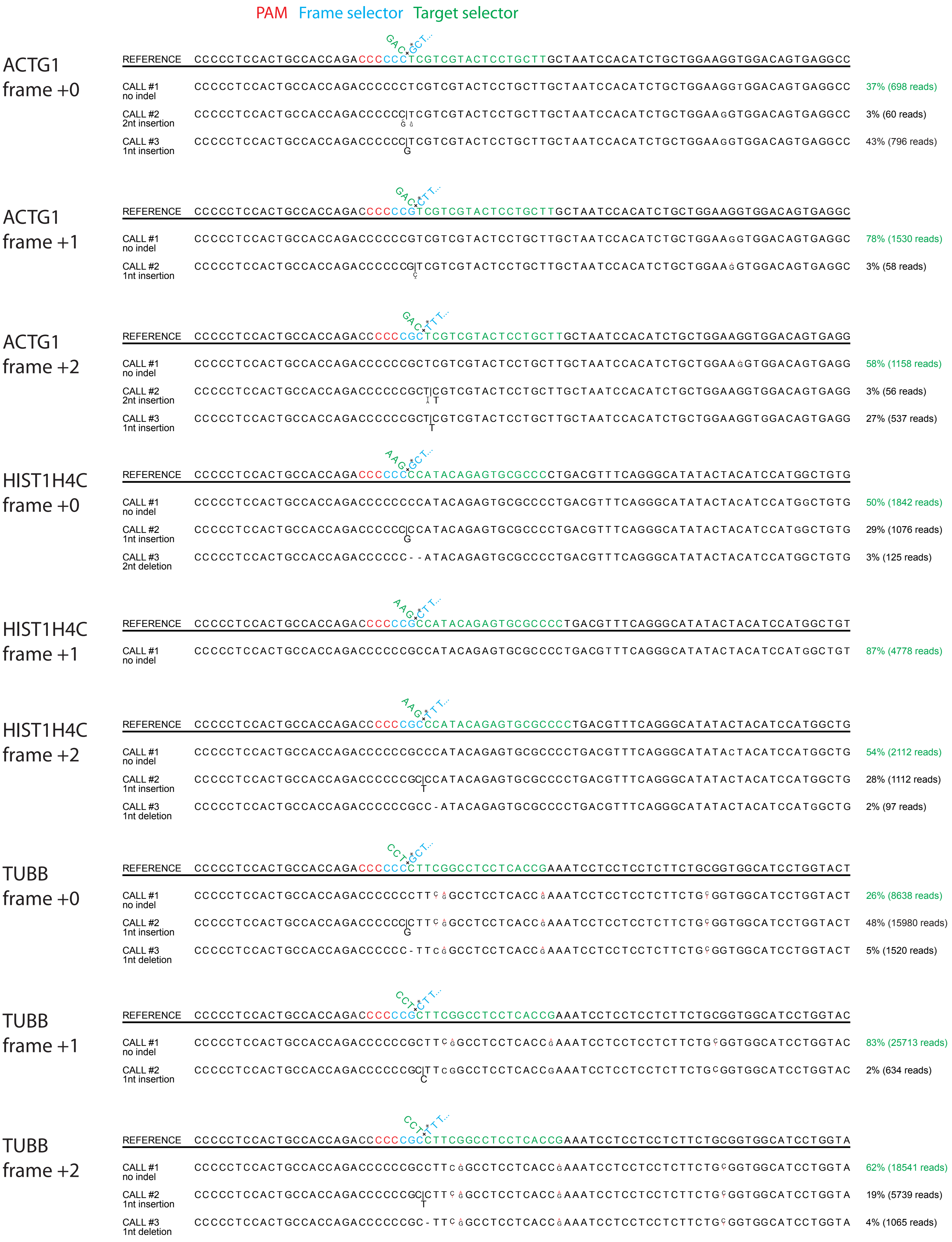

Supplementary Figure 4 | Deep sequencing of NHEJ-mediated fusion events. Shown are alignments of consolidated deep sequencing reads to a seamless integration sequence after CRISPaint-mediated gene tagging using the target- and frame selectors indicated. PAM sequences are marked in red, the frame selector target sites are marked in blue, and the target selector target sites are marked in green. The size of the bases in the alignments linearly correlates with their respective frequency in the sequencing data, with base frequencies <20% being omitted. Indel events with frequencies <2% are omitted. Asterisks denote base -4 with respect to the PAM motif of the frame selector target site. Further explanations are given in Supplementary Note 2.

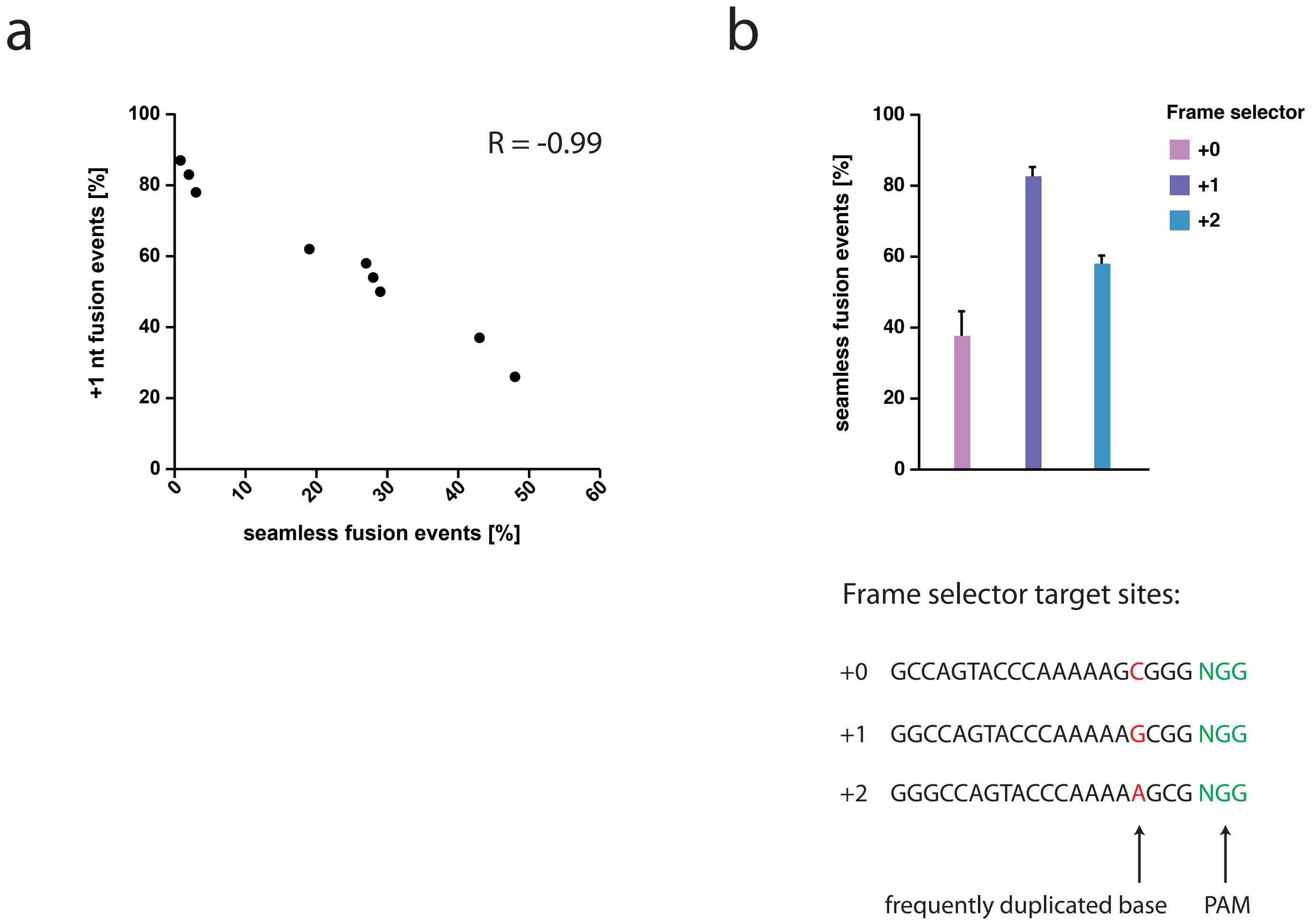

Supplementary Figure 5 | Fidelity determinants of NHEJ-mediated fusion events. (a) Shown are frequencies of single base duplications after NHEJ-mediated gene tagging correlated to frequencies of seamless integration events, both quantified in relation to total integration events via deep sequencing from nine experiments detailed in Fig. 3. R denotes the Pearson correlation coefficient. **(b)** Shown are the mean frequencies of seamless integration events for individual frame selectors +SEM from three gene tagging experiments detailed in Fig. 3. The sequences of the three frame selector target sites are given below. The frequently duplicated base (-4 relative to PAM) and the PAM motif are highlighted in red and green, respectively.

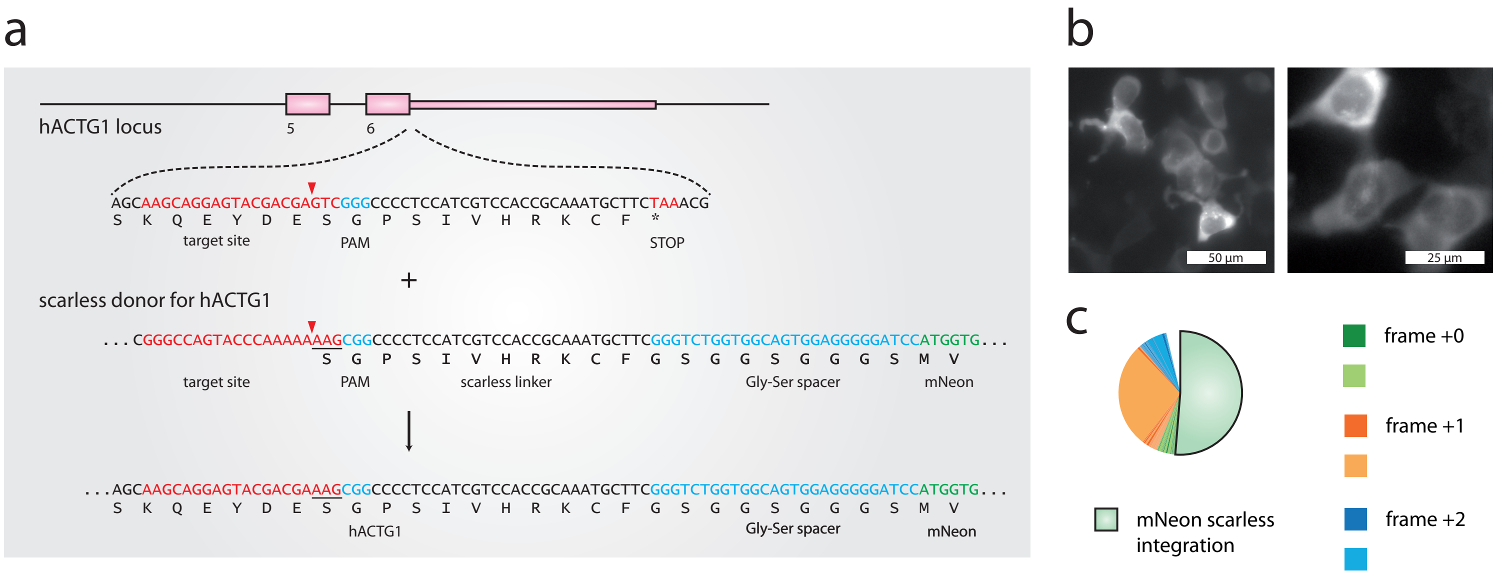

Supplementary Figure 6 | Scarless gene tagging of ACTG1 avoiding loss of C-terminal amino acids. (a) Scheme of the exon structure of the ACTG1 gene targeted by CRISPaint using a donor which re-introduces the amino acids encoded downstream of the genomic target site, allowing scarless gene tagging. **(b)** Microscopic images of HEK293T cells with scarless mNeon-tagging of the ACTG1 gene. **(c)** Deep sequencing analysis of scarless ACTG1-mNeon gene tagging events. Shown are representative data from three biological replicates.

ATM KO

```
REFERENCE   TTAGTGATGCAAACGAACCTGGAGAGAGCCAAAGTACCATAGGTAAATACATATTTACTACTTGGGATTTCTTTTACTTC

CALL #1           TTAGTGATGCAAACGAACCTGGAGAGAGCCAAAGTAC-ATAGGTAAATACATATTTACTACTTGGGATTTCTTTTACTTC   36% (2510 reads)
1nt deletion
CALL #2           TTAGTGATGCAAACGAACCTGGAGAGAGCCAAAGTAC-------------ATATTTACTACTTGGGATTTCTTTTACTTC   22% (1506 reads)
13nt deletion
CALL #3           TTAGTGATGCAAACGAACCTGGAGAGAGCCAAAGTACCA-------ATACATATTTACTACTTGGGATTTCTTTTACTTC   42% (2954 reads)
7nt deletion
```

PRKDC KO A

```
REFERENCE   ACCGCTGCGGTGCTGCCCTGGCCGGTCATCAACTGATCCGCGGCCTGGGGCAGGAATGCGTCCTGAGCAGCAGCCCCGCG

CALL #1           ACCGCTGCGGTGCTGCCCTGGCCGGTCA--------CCCGCGGCCTGGGGCAGGAATGCGTCCTGAGCAGCAGCCCCGCG   36% (90 reads)
8nt deletion
CALL #2           ACCGCTGCGGTGCTGCCCTGGCCGGTCATCAACTGA----------------GGAATGCGTCCTGAGCAGCAGCCCCGCG   36% (89 reads)
16nt deletion
CALL #3           ACCGCTGCGGTGCTGCCCTGGCCGGTCATCAACTGAT--GCGGCCTGGGGCAGGAATGCGTCCTGAGCAGCAGCCCCGCG   28% (69 reads)
2nt deletion
```

PRKDC KO B

```
REFERENCE   ACCGCTGCGGTGCTGCCCTGGCCGGTCATCAACTGATCCGCGGCCTGGGGCAGGAATGCGTCCTGAGCAGCAGCCCCGCG

CALL #1           ACCGCTGCGGTGCTGCCCTGGCCGGTCATCAACTGAT|CCGCGGCCTGGGGCAGGAATGCGTCCTGAGCAGCAGCCCCGCG   100% (708 reads)
1nt insertion                                          T
```

NHEJ1 KO A

```
REFERENCE   AGCAAGGCCTGTTGATGCAGCCATGGGCGTGGCTACAGCTTGCAGAGAACTCCCTCTTGGCCAAGGTTTTTATCACCAAG

CALL #1           AGCAAGGCCTG--------------------GCTACAGCTTGCAGAGAACTCCCTCTTGGCCAAGGTTTTTATCACCAAG   56% (928 reads)
20nt deletion
CALL #2           AGCAAGGCCTGTTGATGCAGCCATGG-CGTGGCTACAGCTTGCAGAGAACTCCCTCTTGGCCAAGGTTTTTATCACCAAG   43% (710 reads)
1nt deletion
```

NHEJ1 KO B

```
REFERENCE   AGCAAGGCCTGTTGATGCAGCCATGGGCGTGGCTACAGCTTGCAGAGAACTCCCTCTTGGCCAAGGTTTTTATCACCAAG

CALL #1           AGCAAGGCCTGTTGATGCAGCCATGG-CGTGGCTACAGCTTGCAGAGAACTCCCTCTTGGCCAAGGTTTTTATCACCAAG   100% (1179 reads)
1nt deletion
```

DCLRE1C KO A

```
REFERENCE   GCTCCATTCTAGCAGCTTCTCCTTGCGCCAATCTGAAGTCTCCTGTGTACAGGACAGTTCCATTATTGCCCTGAAATAAA

CALL #1           GCTCCATTCTAGCAGCTTCTCCTTGC|GCCAATCTGAAGTCTCCTGTGTACAGGACAGTTCCATTATTGCCCTGAAATAAA   37% (332 reads)
1nt insertion                               T
CALL #2           GCTCCATTCTAGCAGCTTCTCCTTGCG|CCAATCTGAAGTCTCCTGTGTACAGGACAGTTCCATTATTGCCCTGAAATAAA   28% (258 reads)
1nt insertion                                G
CALL #3           GCTCCATTCTAGCAGCTTCTCCTTGCG----------GTCTCCTGTGTACAGGACAGTTCCATTATTGCCCTGAAATAAA   34% (311 reads)
10nt deletion
```

DCLRE1C KO B

```
REFERENCE   GCTCCATTCTAGCAGCTTCTCCTTGCGCCAATCTGAAGTCTCCTGTGTACAGGACAGTTCCATTATTGCCCTGAAATAAA

CALL #1           GCTCC--------------------------------GTCTCCTGTGTACAGGACAGTTCCATTATTGCCCTGAAATAAA   29% (265 reads)
32nt deletion
CALL #2           GCTCCATTCTAGCAGCTTCTCCTTGC--CAATCTGAAGTCTCCTGTGTACAGGACAGTTCCATTATTGCCCTGAAATAAA   52% (476 reads)
2nt deletion
CALL #3           GCTCCATTCTAGCAGCTTCTCCTTGCG|CCAATCTGAAGTCTCCTGTGTACAGGACAGTTCCATTATTGCCCTGAAATAAA   17% (155 reads)
1nt insertion                                G
```

LIG4 KO A

```
REFERENCE   CCAATGCTAGCTGCTATTGCAGATATTGAGCACATTGAGAAGGATATGAAACATCAGAGTTTCTACATAGAAACCAAGCT

CALL #1           CCAATGCTAGCTGCTATTGCAGATATTGAGCACAT--AGAAGGATATGAAACATCAGAGTTTCTACATAGAAACCAAGCT   100% (5104 reads)
2nt deletion
```

LIG4 KO B

```
REFERENCE   CCAATGCTAGCTGCTATTGCAGATATTGAGCACATTGAGAAGGATATGAAACATCAGAGTTTCTACATAGAAACCAAGCT

CALL #1           CCAATGCTAGCTGCTATTGCAGATATTGAGCACA|TTGAGAAGGATATGAAACATCAGAGTTTCTACATAGAAACCAAGCT   23% (672 reads)
5nt insertion                                     ACATA
CALL #2           CCAATGCTAGCTGCTATTGCAGATATTGAGCACATTGA|GAAGGATATGAAACATCAGAGTTTCTACATAGAAACCAAGCT   42% (1214 reads)
1nt insertion                                          A
CALL #3           CCAATGCTAGCTGCTATTGCAGATATTGAGCACATTGA--AGGATATGAAACATCAGAGTTTCTACATAGAAACCAAGCT   34% (991 reads)
2nt deletion
```

Supplementary Figure 7 | Genotyping results of KO clones generated using CRISPR/Cas9. HEK-Cas9 monoclones were genotyped using deep sequencing. Data were evaluated using the OutKnocker.org genotyping software. Called alleles with frequencies of >2% are shown. CRISPR target sites are marked in blue.

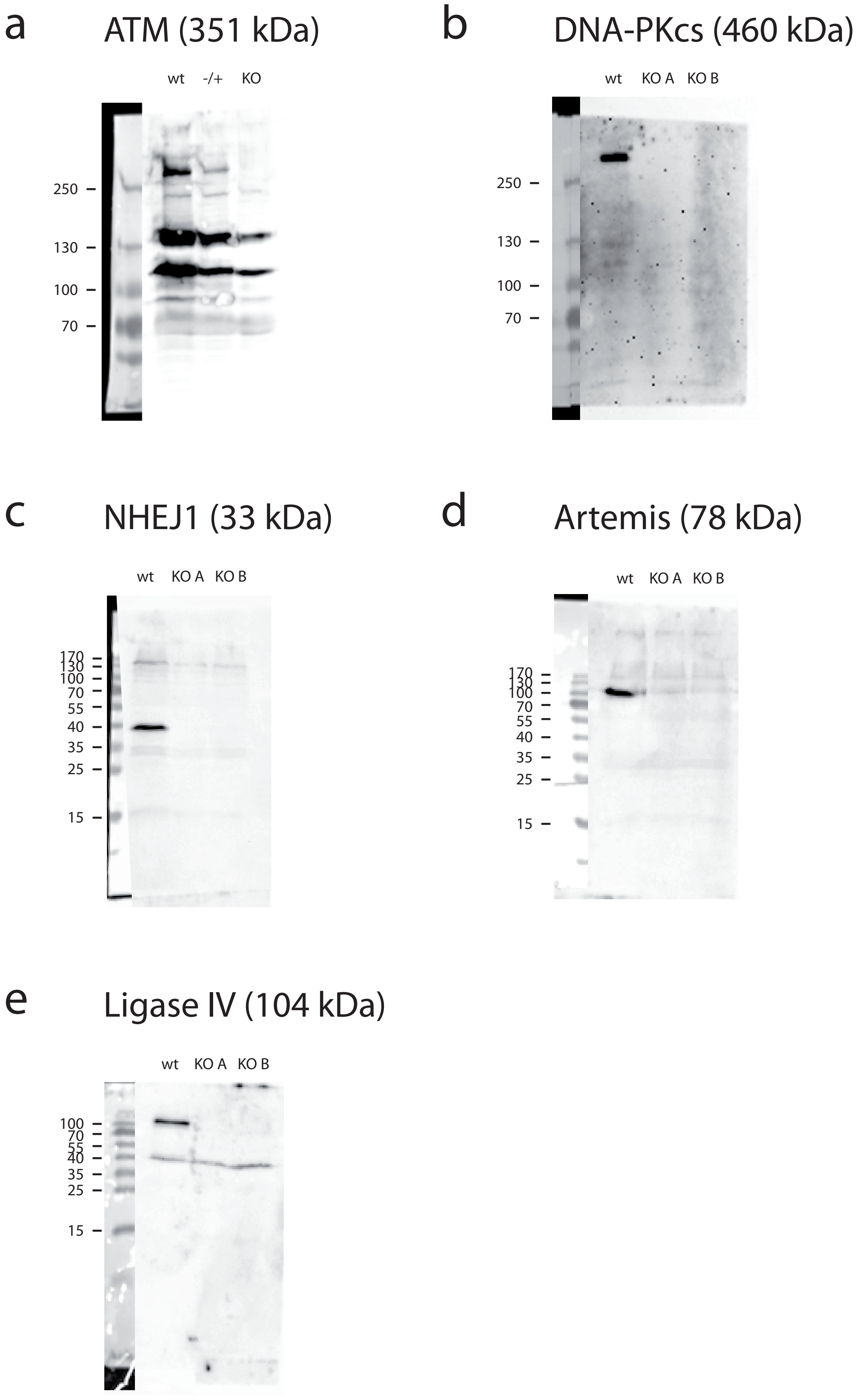

Supplementary Figure 8 | Uncropped immunoblots for knockout clone validation detailed in Fig. 6.
(a) ATM **(b)** DNA-PKcs **(c)** NHEJ1 **(d)** Artemis **(e)** Ligase IV.

Supplementary Note 1 | Target site selection rule based on the PAM-proximal 3-mer sequence

The donor plasmid and the genome are cleaved by the target selector and the frame selector sgRNA at position -3 relative to their respective PAM motifs (NGG). A donor integration event by seamless re-ligation will create a fusion target site in the genome consisting of 17 bases from the original target site and 3 bases (+ PAM motif) from the donor target site. In principle, this fusion target site could be object to re-cleavage by the target selector sgRNA, which would reduce the frequency of perfect in-frame integration of an heterologous ORF, e.g. to create a tagged fusion protein. Re-cleavage is expected to be efficient if the fusion target site closely resembles the original genomic target site, which is the case if the 3 PAM-proximal bases of donor and genomic target site are similar or equal.

In line with this reasoning, we frequently noted that when integrating donors with the PAM-proximal 3-mer sharing more than one base of homology with the PAM-proximal 3-mer of the genomic target site, the fraction of seamless integrants is markedly decreased. As an example, Supplementary Fig. 3 shows deep sequencing results from the integration of an mNeon donor into a genomic locus 3 bases shifted from that used in Fig. 3b (*HIST1H4C*). At this genomic target site, the PAM-proximal 3-mer sequences of donor and genomic target site are identical (underlined). Accordingly, nearly no perfect in-frame fusion events are detected using neither frame selector.

From these observations, we deduced a general rule that a genomic target site must not share more than one base of homology in its PAM-proximal 3-mer with the frame selector sgRNA used. Even though slightly restraining targeting possibilities, an *in-silico* generated human genome-wide CRISPaint targeting library (Supplementary Data 2) removed on

average only 5.4 amino acids from natural protein sequences, while always obeying the above 3-mer rule.

Supplementary Note 2 | Deep sequencing analysis of NHEJ-mediated fusion events

In order to analyze indel mutations within the fusion junctions of CRISPaint gene tagging, PCRs were performed on lysates of CRISPaint-tagged cells using a forward primer binding in the mNeon tag and a reverse primer binding in the respective target locus. Nine experiments (3 frame selectors x 3 target genes) were analyzed in parallel by deep sequencing and data were aligned to hypothetical seamless junction sequences using OutKnocker.org (same experiments as shown in Fig. 3). Herein, a seamless junction was assumed to be a blunt end ligation of the donor and the target locus at a putative Cas9 cleavage site 3 bases upstream of the respective PAM motif.

What can be seen in the results (Supplementary Fig. 4) is that indeed seamless junctions are the predominant sequencing result in most experiments, as it can be appreciated by the high percentages on the right marked in green. However, especially for frame selector +0, a 1nt insertion often makes up a high proportion of insertion events (e.g. *ACTG1*, frame +0: 43%).

The single base that is frequently inserted mostly equals base -4 from the PAM of the frame selector target site marked in blue. Here it is important to keep in mind that the deep sequencing data is antisense to the CRISPR target sites due to the forward primer facing backwards from the tag gene, meaning that the PAM is reverse complemented (CCC marked in red). Base -4 is the forth blue base from left to right in each reference sequence, which is the first base depicted angular relative to the green sequence (marked with an asterisk in Supplementary Fig. 4) due to the fact that it should not be retained in the intended seamless fusion.

This result, albeit merely descriptive, implicates a cryptic molecular mechanism of base duplication, since if the duplication of base -4 of the donor was merely caused by an

infidelity of Cas9 cleavage positioning, one would expect a similarly enriched 1nt deletion of base -4 of the target selector sequence. This, however, is not observed.

At the same time, it is interesting to note that the average fidelity of integration events seems to reproducibly vary among frame selectors (Supplementary Fig. 5b), with frame selector +0 being roughly two times as prone to the -4 base duplication as frame selector +1. The base identity at position -4 differs between the frame selectors, and could hypothetically be the determinant of insertion fidelity. If confirmed by future experiments, this feature would allow a rational re-design of the frame selectors and donors for enhanced tagging fidelity, e.g. by introducing Gs at position -4 (as present in frame selector +1). While the limited sample size precludes us from drawing a definite conclusion, this experimental system could provide further insight into the biology of the cellular NHEJ machinery.

Supplementary Note 3 | Design of a gene-specific scarless donor construct

1. Start with the constant sequence 5'-CGGGCCAGTACCCAAAAA-3'.
2. Add to the 3' end a sequence that encodes for any amino acids that would be lost during CRISPaint gene tagging. To avoid re-cleavage after integrating the donor, change as many bases as possible within the first three bases without altering the encoded amino acids.
3. Add to the 3' end the sequence 5'-GGGTCTGGTGGCAGTGGAGGGG-3'.
4. Convert the sequence to sense and antisense oligonucleotide sequences.
5. Add the 5' overhang 5'-CTAG-3' to the sense oligonucleotide, and 5'-GATC-3' to the antisense oligonucleotide.
6. Have the two oligonucleotides synthesized, anneal them, and ligate them into a universal donor plasmid linearized with BamHI and NheI.
7. The target site for linearizing the donor will be 5'-GGGCCAGTACCCAAAAA-NNN-3', where NNN denotes the first three bases introduced in step 2.
